# Obesity and Cancer: From Systemic Metabolic Reprogramming to Immunotherapy Paradox

**DOI:** 10.3390/metabo16030174

**Published:** 2026-03-06

**Authors:** Guoxiao Han, Shuyu Yuan, Wangui Yu

**Affiliations:** Health Science Center, Yangtze University, Jingzhou 434023, China

**Keywords:** obesity, tumor microenvironment (TME), metabolic reprogramming, immune checkpoint inhibitors (ICIs), adipokines, microbiome, circadian disruption, neuroimmune crosstalk

## Abstract

**Highlights:**

**What are the main findings?**

- Obesity drives tumorigenesis through coordinated metabolic, hormonal, and inflammatory remodeling that precedes and conditions tumor microenvironment (TME) barriers.
- The review proposes a multi-layered framework connecting systemic obesity-driven signals (e.g., insulin–IGF axis, leptin–PD-1 signaling, aromatase expression, histone lactylation) with local TME execution modules (perfusion, ECM, immune exhaustion).

**What are the implications of the main findings?**

- Conventional metrics like BMI are insufficient to capture cancer-relevant obesity exposures. Markers such as leptin/adiponectin ratio, histone lactylation, or checkpoint
- Integrating metabolic intervention (e.g., GLP-1-based agents) with immunotherapy may unlock new combination strategies by reversing pseudo-exhaustion and modifying checkpoint sensitivity.

**Abstract:**

With the global rise in overweight and obesity, excess adiposity has emerged as a modifiable carcinogenic exposure. Beyond energy surplus, obesity establishes a durable pro-tumorigenic baseline through endocrine–metabolic rewiring, chronic low-grade inflammation, and structural/mechanical remodeling of tissues, thereby shaping organ-specific microenvironments that favor malignant transformation and progression. This review integrates systemic metabolic and endocrine alterations with tumor microenvironmental physical barriers, immune reprogramming, and neuroimmune regulation to explain heterogeneity in cancer risk, progression, and treatment response. We propose a stratified assessment framework based on measurable indicators—body composition, inflammatory status, and treatment exposure—to support risk prediction, mechanistic validation, and the design of actionable experimental and clinical strategies.

## 1. Introduction

**Figure 1:**
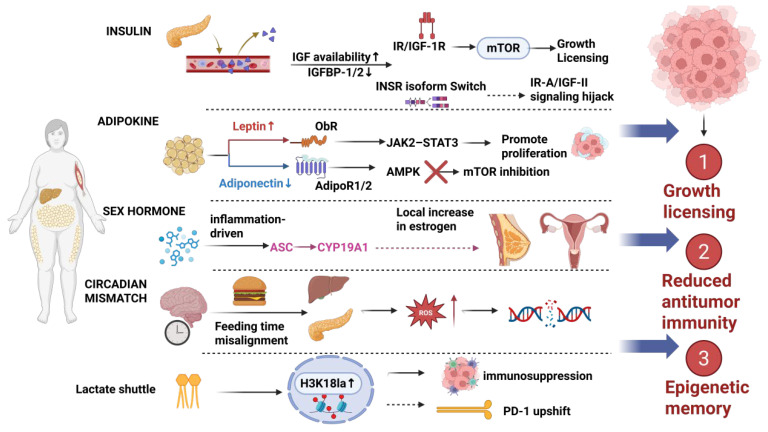
Obesity builds a systemic oncogenic “baseplate” that rewires the metabolic–endocrine milieu and biases tumor progression.

Obesity is not merely a phenotypic manifestation of an imbalance between energy intake and expenditure, but rather a systemic chronic pathological state that spans endocrine, immune, neurohumoral, and tissue biomechanical dimensions [1,2]. Based on abundant epidemiological and mechanistic evidence, the World Health Organization/International Agency for Research on Cancer (WHO/IARC) has defined “excess body fat” as a definite risk factor for at least 13 types of cancer [3]. Large-scale cohort studies have further confirmed the association between obesity and the risk of multiple cancer types, but the risk slopes vary significantly among different cancer types and population subtypes, suggesting that linear extrapolation based solely on BMI has structural limitations [4]. Even in the “metabolically healthy obesity (MHO)” phenotype, the cancer risk remains higher than that of metabolically healthy non-obese individuals, indicating that fat load itself and the accompanying latent inflammation/metabolic perturbations may be involved in the carcinogenic process [5]. Therefore, a mechanistic framework that simultaneously covers the systemic metabolic chassis and the tumor microenvironment execution layer is needed to explain risk stratification, treatment response, and prognostic differences. This review is organized into four parts. First, we describe how systemic metabolic reprogramming provides sustained pro-proliferative signals. Second, we discuss physical and immune barriers in the tumor microenvironment. Third, we summarize cross-organ communication and microbiome-related mechanisms. Finally, we examine boundary conditions and translational implications of the obesity paradox in immunotherapy. To orient the reader, Figure 1 summarizes how obesity establishes a systemic oncogenic “baseplate” through metabolic–endocrine rewiring and durable stress programs, thereby biasing early tumor growth and immune set-points.

Obesity establishes a chronic pro-tumor baseline through converging systemic inputs. Hyperinsulinemia with increased IGF signaling activates the PI3K–AKT–mTOR axis, providing growth licensing for transformed cells. In parallel, adipokine imbalance (leptin↑/adiponectin↓) together with circadian desynchrony elevates inflammatory tone and ROS, shifting immune set-points toward suppression. In hormone-responsive contexts, inflammatory cues induce aromatase (CYP19A1) in adipose stromal cells, increasing local estrogen exposure. Metabolic stress can be translated into chromatin remodeling, exemplified by increased histone lactylation (H3K18la), supporting persistent transcriptional states. Collectively, these inputs drive (1) mitogenic/anabolic licensing, (2) reduced antitumor immunity, and (3) durable epigenetic/metabolic memory.

### Review Scope and Methodology

This manuscript is a narrative review with a framework-building goal: to integrate mechanistic and translational evidence linking obesity-associated systemic remodeling to tumor microenvironment (TME) barriers and heterogeneity in immune checkpoint inhibitor (ICI) response. We conducted targeted literature searches in PubMed, complemented by screening of reference lists from key meta-analyses and landmark clinical trials (between 2006–2026).

Search terms were combined using Boolean operators and included: obesity OR adiposity OR BMI OR visceral fat OR sarcopenia; cancer OR tumor microenvironment; immune checkpoint inhibitor OR PD-1 OR PD-L1 OR CTLA-4; insulin OR IGF OR IGFBP; leptin OR adiponectin; aromatase OR CYP19A1; lactate OR LDH OR histone lactylation; tertiary lymphoid structures; microbiome; circadian disruption; and neuroimmune signaling.

We prioritized (i) meta-analyses, randomized trials, and large multi-cohort studies reporting associations between obesity/body composition and cancer outcomes (risk, progression, or ICI response); (ii) mechanistic animal, organoid, or ex vivo studies providing causal links between obesity-associated cues and TME modules; and (iii) translational biomarker studies with defined assays and multivariable adjustment. Case reports and small uncontrolled series were excluded unless they provided unique mechanistic insights. When findings were contradictory, we reported both directions and interpreted heterogeneity by tumor type, treatment regimen/line, sex, smoking status, cachexia/sarcopenia, and study design.

An operational definition of the oncogenic “baseplate” and testable predictions are summarized in Box 1.

Box 1 **Operational definition of the oncogenic “baseplate” (systemic chassis) and testable predictions.**

Operational Definition

In this review, the oncogenic “baseplate” is operationalized as a persistent, obesity-associated systemic milieu that (i) provides mitogenic/metabolic licensing signals and (ii) shifts immune set-points prior to, and in parallel with, local tumor evolution. Serving as a conceptual synonym for the “systemic carcinogenic chassis,” this baseplate can be quantified using a minimal measurable feature set spanning four modules:

Insulin–IGF availability: Insulin levels, insulin-driven IGFBP-1/2 suppression patterns, and surrogates of free/bioavailable IGF.

Adipokine balance: Leptin-to-adiponectin ratio.

Steroid microenvironment: Menopausal status and aromatase-related proxies (when tissue is available) in hormone-responsive contexts.

Inflammatory/Circadian mismatch: Inflammatory markers (e.g., CRP, NLR) combined with structured exposure indicators (e.g., shift-work timing).

A pragmatic “baseplate score” (0–4) can be computed by assigning 1 point per module if it exceeds a prespecified, cohort-anchored threshold (e.g., upper tertile/quartile cutoffs). Directionality is defined a priori (higher score = higher mitogenic/inflammatory load). This score is proposed as a tool for hypothesis testing and must be rigorously validated before consideration as a clinical decision rule.

Testable Predictions

Prediction 1: BMI-defined “obesity paradox” signals in immune checkpoint inhibitor (ICI) cohorts will attenuate after explicit control for reverse causation (e.g., cachexia) and selection bias. Conversely, a baseplate score incorporating weight trajectories and body composition will retain or improve predictive performance.

Prediction 2: In baseplate-high but barrier-low tumors, PD-1 axis “checkpoint dependence” (pseudo-exhaustion) is more likely to be reversible, associating with an improved response to PD-1/PD-L1 blockade.

Prediction 3: In baseplate-high and barrier-high tumors (e.g., highly fibrotic or TREM2-enriched), ICI benefit will remain limited unless barrier-unlocking strategies are sequentially applied.

Pragmatic Validation Pathway

Phase 1 (Retrospective): Discovery in existing ICI-treated cohorts with standardized baseline multi-omics/blood sampling.

Phase 2 (Validation): Independent external validation across different tumor types and treatment regimens.

Phase 3 (Prospective): Biomarker-enriched prospective studies focusing on pre-specified interactions (Baseplate score × Barrier status × Treatment regimen).

## 2. Systemic Metabolic Reprogramming of the Tumor Microenvironment

The pro-tumorigenic effect of obesity is first manifested at the systemic level through metabolic substrate reprogramming: long-term deviations in blood chemistry, hormone profiles, and inflammatory baselines provide continuous pro-proliferative and anti-apoptotic signals to tissues, while simultaneously altering the threshold of immune regulation [6,7]. The insulin/IGF axis, adipokines, sex hormones, and circadian hormones are not independent but rather amplify each other over a chronic timescale, ultimately shaping a systemic niche that is conducive to tumor initiation and progression [6,8,9]. Overall, the systemic drivers of obesity-related tumors are not determined by a single pathway but rather by the long-term resonance and mutual compensation of multiple endocrine-metabolic pathways. This perspective helps avoid oversimplifying complex phenotypes into a single causal chain and provides a structured entry point for subsequent actionable stratification.

### 2.1. The Insulin–IGF-1 Axis: How Receptors Change and How Signals Are Misused

Hyperinsulinemia/insulin resistance has been repeatedly proposed as one of the key mechanisms underlying obesity-related cancers [6]. Insulin not only participates in glucose homeostasis but is also an evolutionarily conserved mitogenic signaling molecule that provides continuous growth-promoting effects to potential transformed cells through pathways such as PI3K/AKT/mTOR and RAS/MAPK [10,11]. Notably, a “key link” does not equate to a “single dominant force”: inflammation, adipokines, and sex hormones may all play parallel driving roles in different types of cancers and at different stages. Based on its quantifiability and modifiability, this review positions the insulin–IGF axis as one of the most actionable driving forces in the systemic metabolic chassis [6,12].

#### 2.1.1. IGF Availability Issues

In obesity, insulin resistance leads to long-term compensation by pancreatic β cells to cope with insufficient insulin application, resulting in a continuous increase in insulin levels in the blood. Due to the high insulin levels, the liver produces less IGF binding proteins (mainly IGFBP-1/2), causing the IGF system to be more exposed within the body. This process is not linear, and total IGF-1 concentrations do not increase proportionally with insulin levels. In obese or type 2 diabetic patients, the levels of total IGF-1, IGFBP, and free IGF-1 may show different patterns of change, or even opposite patterns [13]. Long-term increases in available or free IGF produce more stable signals, which have a better correlation with risk factors because they can activate the PI3K/AKT/mTOR and RAS/MAPK pathways [10,13]. These pathways work together to stimulate the cell cycle process, prevent cell death, and enhance the ability of cells to move and invade other tissues [7,10].

#### 2.1.2. The Neglected Accomplice of Insulin Receptor Isoform A (IR-A)

Historically, IR has been framed mainly as a metabolic receptor, yet cancer data increasingly support a receptor-level “molecular switch” at the INSR exon-11 splicing step that biases signaling toward the fetal IR-A isoform. In breast cancer, the RNA-binding protein CUGBP1/CELF1 promotes exon-11 exclusion, increases the IR-A:IR-B ratio, and is linked to more aggressive, insulin-responsive phenotypes, providing a concrete mechanism by which tumors can re-tune ligand responsiveness without changing ligand abundance [14]. Mechanistically, IR-A differs from IR-B not only by tissue distribution but also by ligand preference: IR-A binds IGF-II with high affinity, enabling an IGF-II–IR-A mitogenic circuit that can sustain PI3K–AKT and MAPK flux [15]. Importantly, preclinical evidence indicates that INSR activity can confer intrinsic resistance to IGF-1R–targeted therapy, consistent with a bypass route when IGF-1R is inhibited [16]. For obesity-related cancers, the key refinement is not whether this axis exists, but whether the obese metabolic milieu (hyperinsulinemia and associated nutrient stress) increases the likelihood that IR-A-biased receptor states become selected or functionally dominant [17]. Direct, obesity-stratified protein-level validation across cancer types remains limited; therefore, the most defensible wording is that obesity is a permissive and potentially selective background for an IR-A/IGF-II bypass program, rather than a universally proven driver in every tumor. Nonetheless, given the documented role of IR-A in cancer progression and resistance to IGF-1R-specific inhibitors, the IR-A/IGF-II node should be treated as an actionable “signal hijacking” candidate—motivating obesity-stratified quantification of IR-A:IR-B (and hybrid receptors) and rational testing of co-targeting strategies.

### 2.2. The Storm of Adipokines: The Battle Between Leptin and Adiponectin

Adipose tissue is not only an energy storage depot but also a highly active endocrine organ capable of generating hundreds of adipokines [18]. Obesity leads to alterations in the systemic adipokine secretion profile: pro-inflammatory, pro-proliferative, and pro-angiogenic signals are enhanced, while metabolic inhibitory and anti-inflammatory signals are weakened, thus creating a persistent “endocrine noise background”. In tumor biology, this change is typically manifested as elevated leptin levels, decreased adiponectin levels, and an imbalance in their ratio, which continuously activates pathways such as mTOR and STAT3 at the signal transduction level and reshapes the threshold of immune suppression [19,20].

#### 2.2.1. Leptin Balances Inflammation and Immune Responses

In most cases, the level of leptin in the body is positively correlated with body fat. Appetite is also controlled by the amount of body fat, as high fat content leads to an increase in leptin levels in the blood, thereby reducing appetite. Although high concentrations of leptin play a beneficial role in suppressing appetite, its inhibitory effect gradually weakens over time due to the brain’s resistance to it [21,22,23]. The degree of leptin resistance hinders its full function in the body, although other somatic cells, such as immune system cells and cancer cells, can still respond normally to leptin [23,24]. Leptin binds to the Ob-R receptor, initiating a series of physiological responses, including the JAK2-STAT3 pathway, which can promote tumor cell proliferation and metastasis and promote angiogenesis by promoting VEGF [25,26,27]. At the same time, leptin has immunomodulatory properties that can affect T cell differentiation and effector functions, but its direction and magnitude are modulated by the inflammatory background and tissue environment [28,29]. However, prolonged leptin stimulation may also lead to upregulation of PD-1 expression on T cells, thereby causing the cells to enter a negative feedback inhibition state driven by nutritional signals. This metabolic exhaustion is distinct from the common antigen-driven exhaustion and may be partially reversible in some cases, providing an immunometabolic explanation for the obesity paradox [30,31,32].

#### 2.2.2. Key Players in Adiponectin Metabolism

Contrary to leptin, obesity is often associated with a significant decrease in adiponectin (hypoadiponectinemia) [33,34]. Adiponectin activates AMPK through AdipoR1/R2, thereby inhibiting mTOR signaling and promoting fatty acid oxidation. In some contexts, it also enhances autophagy to maintain metabolic homeostasis [33,35,36]. The deficiency of adiponectin means that this “metabolic brake” is released, making mTOR more prone to continuous activation, which not only provides growth permission for tumor cells but also weakens the protective effect of insulin sensitization [37,38].

### 2.3. Sex Hormone Metabolism: Aromatase and the “Estrogen Bath” of the Local Microenvironment

The occurrence and progression of hormone-dependent tumors (breast cancer, endometrial cancer) are closely related to the hormonal environment in the body, which is particularly prominent in postmenopausal women. After the decline of ovarian function, peripheral aromatization (especially in adipose tissue) becomes an important source of postmenopausal estrogen [39]. Adipose stromal cells (ASCs) express aromatase, but expression is dynamic and can be upregulated by inflammatory or tumor-derived cues. For example, such signals can increase aromatase activity and expression in breast stroma [39,40] and activate promoter-specific programs (e.g., promoter I.4) [41,42]. In addition, inflammatory factors can also regulate the expression of aromatase in adipose tissue through various pathways. Across both population- and tissue-level studies, obesity-associated inflammation in breast white adipose tissue (e.g., crown-like structures) often co-occurs with increased local aromatase expression or activity [43]. This pattern supports a mechanistic chain from obesity to inflammation to enhanced aromatization and increased local estrogen exposure. Obesity could influence the depth of estrogen suppression during endocrine therapy by increasing peripheral aromatization and altering the local hormone milieu. However, clinical data do not support BMI as a standalone predictor of aromatase inhibitor benefit. For example, ATAC suggested a stronger relative benefit in the low-BMI subgroup [44], whereas BIG 1–98 did not find a significant interaction between BMI and letrozole benefit over tamoxifen [45], and ABCSG-12 also reported treatment- and population-dependent effects [46]. Therefore, at this stage, it is not advisable to infer the decline in AI efficacy solely based on BMI. A more reasonable approach is to conduct fine stratification by combining menopausal status, compliance, dose exposure, and body composition distribution (for example, compliance/early discontinuation is related to outcomes: [47]. Besides estrogen, androgen metabolism may also be involved in the endocrine shaping of obesity-related tumors: WHI metabolomics shows that in obese women (BMI ≥ 30), waist-to-hip ratio (WHR) is positively correlated with active androgens and their 5α-reduced metabolites (including DHT, etc.) [48]. It is important to note that androgens may not only act as precursors of estrogen to influence local substrate supply but also drive proliferation and resistance-related programs through AR signaling in some breast cancer models or specific molecular backgrounds. In endometrial tissue/endometrial cancer, the role of the AR pathway is more context-dependent, with both risk-related clues and reports of anti-proliferative/differentiation regulation, and thus it should not be generally classified as “pro-proliferative” [49]. Overall, obesity-related steroid metabolic rearrangements are more likely to jointly shape the biological basis of hormone-driven tumors through the coupling of “precursor supply (substrate) × local inflammation—aromatization (output) × receptor signaling threshold (response)”.

### 2.4. Circadian Rhythm Disruption: Systemic Mismatch of Molecular Clocks

The resurgence of obesity is attributed to lifestyle changes that disrupt circadian rhythms, which are now emerging as a factor influencing overall health. Molecular clocks play a crucial role in controlling sleep–wake cycles and maintaining tissue health by regulating metabolic processes, DNA repair, and immune responses. When the biological clock functions are disrupted or chronically misaligned, the body undergoes significant stress, leading to the development of cancer. This section will explore how circadian rhythm disruption, as a systemic imbalance factor, links obesity to differences in tumor risk and treatment outcomes.

#### 2.4.1. The Disruption of Molecular Clocks

Obesity is associated with abnormal expression of peripheral clock genes and weakened rhythmic signals, and is accompanied by metabolic disorders. Against this backdrop, oxidative stress and genomic stability pressure may increase, thereby raising the risk of DNA damage [50,51].

In some models, BMAL1/CLOCK interacts with cell cycle checkpoints (c-MYC, p53), and circadian rhythm disruption may lift growth inhibition and promote tumor development [52]. The relationship between the molecular clock and cancer is highly tissue- and context-dependent. In some models, BMAL1/CLOCK shows tumor-suppressive features, whereas in other settings it may support tumor-cell survival and metabolic adaptation. Therefore, it is not appropriate to consider it a universal tumor suppressor gene [53]. Night shift work is associated with an increased risk of multiple cancers, so when determining its association with cancer, other factors that workers are exposed to during work, such as sleep deprivation, lifestyle habits, and metabolic disorders, should be carefully controlled [54,55,56]. The reduction in the amplitude and phase shift of the biological clock caused by a high-fat diet (HFD), as well as oxidative stress and metabolic load, can regulate DNA damage response and cell cycle control [52,57].

#### 2.4.2. Systemic Mismatch

The key consequence of rhythm disorder is not merely “a change in gene expression”, but rather the systemic mismatch caused by the desynchronization of the central clock (suprachiasmatic nucleus, SCN) and peripheral clocks (liver, pancreas, adipose tissue). When feeding and activity are chronically misaligned, insulin secretion and tissue uptake can become temporally mismatched. This mistiming can raise metabolic inflammation and oxidative stress and reinforce an ‘obesity–circadian suppression–metabolic damage’ feedback loop [58]. The timing of food intake itself can serve as a rhythm synchronization signal; in breast tumor models, restricting food intake to inappropriate time windows disrupts activity and body temperature rhythms and increases tumor growth efficiency [59,60]. As the circadian rhythm and immune axis is a field with practical translational application value, the timing of drug administration may affect the application of immunotherapy in clinical practice [61,62,63]. Overall, obesity is interconnected through four systemic pathways, leading to a gradual increase in tissue growth permissibility and the baseline state of immune suppression. These four pathways include the insulin–IGF axis, adipokine profile, steroid hormone microenvironment, and rhythm disorder. Table 1 consolidates these four systemic drivers into measurable readouts and pragmatic stratification handles, enabling exposure-intensity and reversibility assessment before the downstream TME execution layer is considered. In the hospital environment, decisions are not only based on whether obesity increases the risk of cancer, but also on the process and magnitude of changes in these four biological indicators, which helps to identify the intensity of exposure and its reversibility, thereby determining the appropriate treatment window and combination therapy. Importantly, insulin–IGF signaling, adipokines, steroid hormones, and circadian mismatch do not act only at the circulating level. They are ultimately translated into local, observable TME phenotypes—perfusion deficits and hypoxia, ECM remodeling, myeloid recruitment, and immune–metabolic threshold shifts, extracellular matrix (ECM) remodeling, myeloid cell recruitment, and immune metabolic threshold resetting. Based on this execution logic, the next chapter will focus on how mechanical–immune–neural interactions within the TME shape tumor progression and treatment response.

## 3. Physical Barriers and Immune Remodeling in the Tumor Microenvironment (TME)

**Figure 2:**
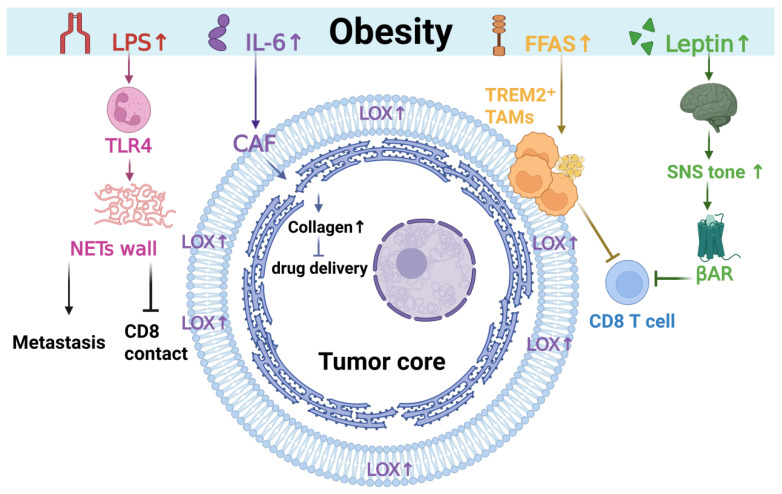
Obesity constructs coupled physical and immune barriers that restrict drug delivery and CD8 T-cell access in the tumor microenvironment.

Obesity not only alters the body’s signaling system but also fundamentally changes the environment in which tumors grow. This chapter will explore the interactions among physical mechanics, immune cell subsets, and the nervous system within the tumor microenvironment. While systemic drivers provide growth and inflammatory licensing, their clinical consequences are largely executed locally as coupled physical and immune barriers; Figure 2 depicts this barrier architecture that constrains drug penetration and CD8 access in the TME.

Obesity-associated cues (LPS↑, IL-6↑, FFAs↑, and leptin↑) converge on the tumor niche to build an “exclusionary” microenvironment. LPS activates TLR4-dependent neutrophil programs and promotes a NETs wall, which facilitates metastasis and reduces effective CD8 contact. IL-6 supports CAF activation and enhances LOX-associated collagen deposition/crosslinking, increasing matrix stiffness and limiting intratumoral drug delivery. Lipid-rich signals recruit or sustain TREM2^+^ tumor-associated macrophages that reinforce fibrotic remodeling and immunosuppression. In parallel, leptin-driven sympathetic tone (SNS↑) augments β-adrenergic signaling in CD8 T cells, dampening effector function. Together, these intertwined stromal–myeloid–neural modules form a barrier architecture that shapes treatment penetration and antitumor immunity.

### 3.1. Mechanobiology: Stiffening of the Matrix and Mechanical Memory

The excessive expansion of adipose tissue is not merely an increase in volume but also a profound biomechanical remodeling that profoundly affects the fate of tumor cells.

#### 3.1.1. Fibrosis and Stiffening of the Extracellular Matrix (ECM)

In obesity, adipocyte hypertrophy and insufficient perfusion can push tissues into a self-reinforcing ‘hypoxia–inflammation–fibrosis’ loop [77]. This remodeling increases collagen deposition and alters matrix architecture, forming a physical barrier around tumors [78,79,80]. The actual stiffness of adipose tissue is highly dependent on the measurement method (atomic force microscopy, ultrasound/magnetic resonance elastography, etc.) and the type of adipose tissue sampled (subcutaneous fat or visceral fat) [79,81,82]. Therefore, it is more appropriate to focus on the directional increase and significant heterogeneity of the system’s growth trend rather than attempting to infer a single threshold point. When the ECM stiffens, integrin-based adhesion complexes and the FAK–RhoA–actin tension axis transmit mechanical cues to the nucleus. This signaling promotes YAP/TAZ nuclear localization and transcriptional reprogramming, which can enhance proliferation, survival, and migration [83,84]. In fact, this mechanical remodeling is not only beneficial to tumor cells: myeloid cells can also participate in the co-shaping process of fibrosis and immune rejection. For example, collagen deposition by myeloid cells may occur simultaneously with CD8+ T cell dysfunction, ultimately leading to the formation of a permanent immune barrier [85,86]. Therefore, this section proposes to describe ECM hardening as a measurable, hierarchical microenvironment state variable that acts as a multivariate influence on drug delivery, immune infiltration, and tumor progression, rather than a simple histological finding [87,88]. If collagen cross-linking or tissue elasticity is used as a stratification axis, YAP target genes (e.g., CTGF and ANKRD1) and T-cell exclusion should show threshold-like changes. In highly stiffened tumors, targeting the LOX/FAK axis or applying anti-fibrotic strategies should improve perfusion, facilitate T-cell entry, and enhance subsequent immunotherapy responses [86,89].

#### 3.1.2. Residual Risks of Mechanical Memory After Weight Loss

Mechanical memory describes the persistence of mechanically induced transcriptional states after mechanical unloading. After prolonged exposure to high stiffness or tension, YAP/TAZ-linked programs may decay slowly and remain partially active even when the external matrix softens [90]. The key point of this concept does not lie in “whether it exists or not”, but rather in the fact that the intensity and duration of mechanical preconditioning, the type of cell, and the chromatin state collectively determine whether it is a transient residue or a stable fixation. Therefore, it is not appropriate to cover all tissues and cancer types with a single conclusion [91,92]. In tumors, mechanical memory can be framed as one candidate layer explaining ‘residual risk after weight loss.’ Persistent YAP/TAZ-linked modules, adhesion programs, or stress-adaptation states may limit full reprogramming to a softer post–weight-loss microenvironment. adhesion/migration programs, or stress adaptation. Consistent with this view, obesity can also leave detectable epigenetic “metabolic memory” at the tissue level, manifested as the persistence of some transcriptional/chromatin features after weight loss, suggesting that “exposure withdrawal does not immediately reset the molecular state to zero” [91]. If mechanical preconditioning exceeds a reversibility threshold, residual YAP/TAZ nuclear localization and selected chromatin marks may persist after weight loss. In this ‘residual-phenotype–positive’ subgroup, short-term anti-fibrotic therapy or YAP/TAZ-pathway inhibition should yield clearer risk-mitigating effects [93].

The biophysical basis of mechanical memory is not only the accumulation of YAP/TAZ in the cytoplasm, but also the structural remodeling of the cell nucleus. The latest bioengineering models show that long-term exposure to high-hardness matrices (such as fibrotic adipose tissue in obesity) leads to a sustained increase in nuclear membrane tension [93]. To resist this external mechanical pressure, tumor cells significantly upregulate the expression and phosphorylation of nuclear lamina protein Lamin A/C [94]. High levels of Lamin A/C not only reinforce the nuclear membrane but also directly alter the topological structure of chromatin (TADs), keeping pro-fibrotic and pro-metastatic gene loci in an open and active state [93]. Chromatin conformational changes mediated by Lamin A/C can persist for weeks to months even after cells leave a stiff matrix [95,96]. This persistence may occur during metastasis to softer tissues or after rapid tissue softening induced by surgery or medication. This “nuclear matrix stiffness” constitutes a physical “epigenetic scar”, explaining why tumor cells retain an invasive phenotype even after weight normalization. Therefore, treatment strategies should not rely solely on weight loss and may need to incorporate drugs targeting nuclear mechanical signaling (such as histone deacetylase inhibitors or nuclear export protein inhibitors) to “erase” this mechanical memory.

### 3.2. Immune Microenvironment: Paradigm Shift from M1/M2 to LAMs

A core change in obesity-related immune remodeling is that the M1/M2 model is no longer applicable [97,98]. New research indicates that tumor-associated macrophages (TAMs) in fat and tumors are more like a combination of continuous spectra and functional modules rather than a choice between two mutually exclusive options [99,100,101]. Based on this spectrum structure, the state of macrophages is usually determined by metabolic stress and the surrounding microenvironment. In a single cell, lipid metabolism, phagocytic/lysosomal activity, extracellular matrix modification, and immunosuppressive gene expression programs can occur simultaneously, ultimately reaching a steady state known as metabolic-structural-immune coupling [102,103]. Therefore, the writing style of this section should shift from “labeling” to “describing modules”: focusing more on which myeloid modules’ alteration directions are consistent with tumor growth or immune therapy resistance, and whether these alterations are more common in an obese state. Obesity leads to abnormal increases in immune cell numbers and changes in their structure. Some myeloid cells are more likely to aggregate around scar bands, damaged blood vessels, or T-cell exclusion sites, which affects the way effector cells enter and reside in the body [85,104]. Based on this logic, when discussing lipid-associated macrophages (LAMs) or the TREM2 axis later, it is necessary to actively distinguish which evidence belongs to the “phenotype-related” transcriptome and which has causal support for functional intervention. In spatial transcriptomics or multiplex imaging, the lipid-processing TAM module (e.g., TREM2/LPL/CD36) should co-localize with collagen-rich bands and T-cell exclusion zones. If this module is inhibited or reprogrammed, fibrosis relief and increased ICI sensitivity should be observed in parallel [102,105,106].

#### 3.2.1. TREM2+ Lipid-Associated Macrophages (LAMs)

TREM2^+^ lipid-associated macrophages (LAMs) illustrate a ‘metabolic myeloid module.’ They express high TREM2 and lipid-uptake genes (e.g., Lpl, Cd36, Fabp4) and often form crown-like structures around dying adipocytes, consistent with a response to lipid spillover and tissue damage [107]. In the context of simple obesity, LAMs play a role in “damage control/lipotoxicity buffering” by consuming and processing lipids, thereby limiting lipid diffusion [107,108]. In the tumor microenvironment, LAM-like/TREM2^+^ macrophages promote immune tolerance by suppressing immune responses and promoting T cell exclusion [109,110]. It is essential to separate phenotypic correlations from causal mechanisms. Lipid-metabolism-related markers such as SPP1 may point to roles in ECM remodeling and immune-architecture reorganization, but whether they measurably compete for fuels required by effector cells remains to be tested using metabolic tracing and spatial causal experiments. Notably, interventions targeting the TREM2 axis have stronger causal evidence: blocking/targeting TREM2 can alter the state of tumor-associated myeloid cells and enhance the efficacy of PD-1/PD-L1 inhibitors, which is a unique and reversible key point in combination therapy [111,112]. In obesity-associated tumors, enrichment of a TREM2^+^ TAM/LAM-like program may co-occur with fibrosis and immune exclusion, contributing to primary ICI resistance. Accordingly, ‘high TREM2 expression plus collagen enrichment’ could predict greater benefit from anti-TREM2 combined with anti-PD-1, whereas benefit may be smaller in low-TREM2 or weakly fibrotic subgroups [105].

#### 3.2.2. Neutrophil Awakening: NETs Build Highways for Metastasis

Under the background of obesity, in addition to macrophage-mediated chronic inflammation, the neutrophil-NET axis has been repeatedly observed to be associated with enhanced metastatic phenotypes, especially in the pre-metastatic niches of distant organs such as the lungs [113,114,115]. Obesity promotes the expansion of circulating myeloid cells and enhances neutrophil-related chemotaxis and activation programs through the adipose tissue inflammation and bone marrow myeloid production signaling axis [115,116,117]. Elevated free fatty acids (FFAs) in the blood of obese patients and lipopolysaccharide (LPS) from intestinal leakage are potent agonists for inducing the formation of neutrophil extracellular traps (NETs) [118]. The components of NETs include neutrophil elastase (ELANE), myeloperoxidase (MPO), and decondensed chromatin DNA and histones. One study showed that circulating tumor cells (CTCs) express the transmembrane protein CCDC25, which specifically interacts with the DNA backbone of NETs [119]. This binding acts like a physical fastener, fixing CTCs in the capillaries of the liver and lungs. It also enhances their motility and invasiveness by activating the ILK-β-parvin signaling pathway within cancer cells. In non-alcoholic fatty liver disease, hepatic sinusoids are occupied by neutrophil extracellular traps (NETs). This NET-rich liver microenvironment creates an ideal pre-metastatic microenvironment [120], significantly accelerating liver metastasis and the colonization of colorectal and pancreatic cancers. NETs not only capture cancer cells but also form a physical barrier around tumors, preventing natural killer (NK) cells and cytotoxic T cells (CTLs) from contacting and killing tumor cells. Histones in NETs can also directly induce T cell exhaustion. In various metastasis models, NET degradation (such as DNase I) or inhibition of NETosis can reduce the metastatic burden; meanwhile, obesity can enhance the myeloid-neutrophil-NET-related axis and is associated with enhanced metastasis. Therefore, combined interventions targeting NETs are worth further validation in obese-related tumor populations [114,121].

### 3.3. Lactate Shuttling and Histone Lactylation: Epigenetic Executors of Metabolic Reprogramming

Traditionally, lactate was regarded as a by-product of the Warburg effect, leading to microenvironmental acidification [122]. However, recent studies have revealed that lactate is a key signaling molecule that links cellular metabolism and gene expression [123,124]. In the context of obesity, hyperglycemia and local hypoxia result in elevated lactate concentrations in tumors and adipose tissues [125]. Histone lysine lactylation (Kla) provides an operational mechanism for how glycolytic lactate accumulation is translated into transcriptional programs; under the high glycolytic/hypoxic load associated with obesity, this pathway may be amplified, thereby affecting the phenotypic stability of tumor cells or immune cells. The discovery of histone lysine lactylation explains how obesity stabilizes the cancer cell state [124,126]. The current model suggests that lactate can be converted into lactoyl-CoA and other donors and undergo lysine lactylation modification under the mediation of transferase [127]. The hypoxic environment associated with obesity leads to excessive production of lactate dehydrogenase A (LDHA) through the HIF-1 pathway, thereby accelerating the conversion of pyruvate to lactate. The accumulated intracellular lactate is transported out of the cell to the extracellular acidic environment through the MCT4 transporter [127]. Meanwhile, lactate, as a nuclear substrate, is catalyzed by histone acetyltransferase p300 (as a “writer enzyme”) to undergo lactylation modification at specific sites on histone H3 and H4, including H3K18la [127]. Similar to acetylation, histone lactylation is usually associated with transcriptional activation and, in specific contexts, tends to drive gene modules related to tissue repair, angiogenesis, and immunosuppression [73]. However, unlike acetylation, lactylation modification selectively activates gene programs related to tissue repair, fibrosis, and immunosuppression. In macrophages, lactate accumulation can increase the levels of histone lactylation such as H3K18la and enhance the transcriptional output of repair/angiogenesis-related genes such as Arg1 and Vegfa, thereby promoting a more pro-repair and immunosuppressive functional state [73].

In the context of obesity and tumor-related conditions, the increase in glycolysis and lactate load can create a persistent lactate pressure on the side of immune cells and drive lactate-related epigenetic remodeling [128]. Before tumor development, myeloid cells in obese individuals may already be programmed to exert immunosuppressive effects due to their genetic makeup [129]. In the scenario of obesity-related NSCLC, the transcriptome indicates enhanced glycolysis and increased lactate load; experimentally, lactate can be transported into T cells and induce histone lactylation, thereby upregulating Pdcd1 (PD-1) expression and promoting an exhaustion-related phenotype [74,130,131]. The elevation of PD-1 levels is not only due to antigen loss but also a result of metabolic changes, which make patients more susceptible to anti-PD-1 therapy [128].

### 3.4. The Neuro–Immune–Tumor Axis: An Overlooked Regulatory Network

The role of the nervous system in tumor biology is receiving unprecedented attention [132]. Obesity profoundly alters the neural innervation patterns of tissues [133].

#### 3.4.1. “Denervation” of Adipose Tissue and “Neurogenesis” in Tumors

White adipose tissue (WAT) undergoes changes during obesity, with alterations in its chemical and immune activity and damage to nerve endings [133]. This damage leads to a reduction in the number of nerve fibers and a decrease in the strength of neural signals, a condition now known as adipose neuropathy. WAT has a clear sympathetic “neuro-adipose” functional connection that drives lipolytic output, so the weakening of neural input is not a bystander variable but may alter the inflammatory-lipolytic coupling mode and local homeostasis of adipose tissue [134,135]. Multiple solid tumors exhibit higher nerve fiber density, and there is currently a lack of direct evidence to establish a causal link between “obesity-related WAT denervation” and “tumor neurogenesis/neural invasion”; rather, they may undergo parallel changes through systemic neuro-immune regulation [132]. At the level of immune effectors, norepinephrine (NE) can limit the proliferation, cytokine release, and killing program of CD8^+^ T cells through β-adrenergic receptors (βARs), thereby weakening the therapeutic efficacy of checkpoint inhibitors (ICIs) [136,137]. In the context of chronic βAR signaling activation, the βAR axis has been proposed as an intervention target to lift the inhibition on CD8 antitumor function and enhance the response to immunotherapy [137]. In mouse models, β-receptor blockade can alleviate catecholamine-mediated immunosuppression and enhance the efficacy of checkpoint inhibitors [136,137]. If βAR inhibition is the main factor, then the higher the tumor NE/βAR transcriptional signature or circulating catecholamine levels, the stronger the recovery of CD8 effector molecules and tumor suppression should be when β-receptor blockers are combined with PD-1/PD-L1 blockade.

#### 3.4.2. Neural-Tumor Synapses and Metabolic Hijacking

In addition to soluble neurotransmitter signaling, synapse-like connections between neurons and tumor cells can form a broader band of neural input circuits, converting neural activity into tumor growth signals [132]. In gliomas, tumor cells can receive glutamatergic synaptic inputs from excitatory neurons and then promote cell proliferation, drive invasion, and expand the tumor network through AMPA receptor-mediated electrophysiological coupling. Both animal experiments and human samples consistently support this mechanism [138]. Brain metastases and other tumors also exhibit pseudo-synapses or pseudo-synaptic structures [139,140]. In pancreatic ductal adenocarcinoma, there are glutamatergic pseudo-synapses between sensory nerves and tumor cells, which increase tumor growth and metastatic potential [141]. These studies collectively present a practical model in which tumors can externalize traditional synaptic modules and utilize neural excitation to gain metabolic and invasive advantages. However, the universality of this phenomenon across various cancer types and anatomical locations still requires further stratified experimental verification [132]. A critical review should avoid considering this mechanism as a universal operating principle for all solid tumors and instead focus on the more compelling evidence in central nervous system tumors, brain metastases, and highly neuroinvasive tumors [132]. If synaptic input is a dominant driver, lesions with higher levels of synaptic markers (e.g., synaptophysin and PSD95) and stronger NMDAR/AMPAR activity should be more sensitive to receptor antagonists or interventions that disrupt neuron–tumor contact. This prediction can be tested using electrophysiology together with spatial imaging.

## 4. Intercellular Communication Across Organs and Emerging Mechanisms of Carcinogenesis

Obesity not only leaves epigenetic “scars”, but also continuously transmits carcinogenic signals through the cross-organ metabolic communication network (especially the gut-liver axis) and extracellular vesicles, and reshapes the death fate of cells.

### 4.1. Epigenetic Memory: Scars on Chromatin

Weight loss can significantly improve body fat load and some metabolic indicators, but an increasing number of human multi-omics data suggest that adipose tissue does not always simultaneously “reset” the transcriptional and chromatin programs formed during the obese period. Single-nucleus transcriptomic and ATAC-seq studies suggest that adipocyte shrinkage after bariatric surgery can outpace the resolution of inflammation- and fibrosis-related regulatory programs [91,142]. This persistence is consistent with an ‘obesogenic memory’ state that may prime adipose tissue for reactivation under renewed metabolic stress. Epigenetic memory is the most appropriate description of such residues, and the main factor is not permanence but rather the balance between the rate of clearance and the frequency of external reapplication. Meanwhile, more persistent indicators, such as DNA methylation, may enhance the upper limit of gene expression and retain a longer risk tail in some weight loss populations [143,144]. The reduction in risk after weight loss depends on reversible memory, the duration of memory, and additional metabolic shocks. If the main cause of residual risk is epigenetic memory, then the levels of residual inflammatory or fibrotic activity in exosomes or adipose tissue will become reliable indicators of the rate of weight gain or tumor-related inflammatory markers.

### 4.2. Gut Microbiota: Metabolites as Carcinogenic Messengers

The dysbiosis of the gut microbiota associated with obesity should not be solely measured by the increase in the Firmicutes/Bacteroidetes (F/B) ratio, as this ratio varies greatly among different studies, diets, and analytical techniques, which may lead to erroneous conclusions [145,146]. A more universal approach focuses on functional impacts: gut bacteria continuously regulate inflammation and epithelial stress through pathways such as bile acid transformation, short-chain fatty acid production, endotoxin exposure, and carcinogenic toxin generation [147,148]. These metabolite–immune pathways may contribute differently across cancer types. In the intestine, barrier disruption and mucosal inflammation are central to colorectal carcinogenesis and are influenced by microbiota-derived bile acids [147]. In NAFLD–HCC, longer-term bile-acid signaling and liver–immune crosstalk are thought to shape a pro-tumor inflammatory milieu [149]. The signals from the gut microbiota are not unidirectional pro-cancer signals; their direction changes with the host’s dietary status, barrier integrity, and immune status. If functional effectiveness is more important than microbial composition, then constructing functional fingerprint profiles of bile acid spectra, short-chain fatty acids, and plasma lipopolysaccharides will be more effective in predicting tumor-related inflammation and the immunotherapy sensitivity of obese patients than the F/B ratio.

#### Colonization and Translocation of Pathogenic Bacteria

Regarding obesity-related inflammation, certain oral or intestinal bacteria are more likely to gain colonization and expansion advantages and promote tumor formation/growth. Fusobacterium nucleatum is a typical example: its FadA adhesin can bind to E-cadherin on epithelial cells and activate β-catenin signaling, thereby driving inflammatory and oncogenic transcriptional programs, providing a direct mechanistic link for its pro-cancer phenotype in colorectal cancer [150]. Another classic pathogenic axis comes from enterotoxigenic Bacteroides fragilis (ETBF): in tumor-susceptible models, BFT can trigger a multi-step pro-inflammatory cascade dependent on IL-17R/NF-κB/STAT3 and promote colon tumorigenesis [151]. For pks+ toxin-producing Escherichia coli, the colibactin it releases not only induces DNA damage and mutations, but also its related mutation signature is recognizable and has etiological significance in colorectal cancer genomes [152,153]. Obesity can be accompanied by impaired intestinal barrier function and increased permeability, and is paralleled by a reduction in some barrier-protective commensal bacteria, which may lower the threshold for exposure of pathogenic bacteria and their products across the barrier [145,148]. Obesity itself does not “produce” these pathogens, but rather is more likely to provide them with a relative advantage through barrier disruption, alteration of immune thresholds, and reconfiguration of ecological niche competition [145,148]. The FadA and BFT axis points to potential nodes of pathogenic mechanisms that can be intervened; whether it can bring higher net benefits in the context of obesity stratification still needs to be directly verified in stratified animal models and clinical cohorts.

Microbial metabolic products are the most easily transported signal carriers between organs in the obesity-cancer axis, but their effects are highly context-dependent. Butyrate provides a useful example. In normal colonocytes, butyrate is largely oxidized as an energy source; in Warburg-like metabolic states its oxidation is limited and intracellular butyrate can accumulate. Under those conditions, butyrate can act as an HDAC inhibitor to reshape transcription, producing biological effects that differ from—and may oppose—its role as a fuel [154]. Secondary bile acids (e.g., deoxycholic acid, DCA) can be enriched under obesity-associated microbiome shifts. In the liver, bile-acid signaling intersects with inflammation, fibrosis, and immune homeostasis and may contribute to the evolution from NAFLD to HCC [149]. It is also suggested to supplement the classic mechanism chain to support the causal closed loop of “obesity-DCA-promoting liver cancer” [155]. On the other hand, obesity-related barrier disruption can allow low levels of lipopolysaccharide (LPS) to continuously enter the circulation and form metabolic endotoxemia, thereby amplifying systemic inflammation and metabolic abnormalities [148]. The gut microbiome can modulate immunotherapy response. Cohort studies and systematic reviews report that antibiotic exposure—often a proxy for dysbiosis—is associated with poorer ICI efficacy or survival, but causality and residual confounding require prospective clarification [156,157]. Furthermore, some commensal bacteria can serve as clinical stratification signals: for instance, Akkermansia muciniphila has a predictive association with the clinical response to PD-1 blockade therapy [158]. Therefore, a more reliable translational conclusion is not that a certain metabolite is necessarily carcinogenic or anti-carcinogenic, but that these metabolic inputs can alter the host’s inflammatory threshold and immune homeostasis, making tumor evolution more inclined towards specific immune niches. If functional metabolite fingerprints are a key driver, one testable translational hypothesis is that targeting bile-acid pathways or endotoxin burden will shift inflammatory readouts and biomarkers linked to ICI response. This should be evaluated in stratified cohorts and, ideally, in intervention studies.

### 4.3. Distant “Cargo” Transport by Exosomes

Human organs communicate through multiple pathways to establish the tumor immune microenvironment, and these pathways are not limited to obesity-related soluble hormones and inflammatory factors. The tumor microenvironment can also receive distant signaling pathways in the form of extracellular vesicles (EVs) released from adipose tissue. These vesicles have targeted delivery capabilities and can regulate the tumor microenvironment at the metabolic and immune levels [80,159]. Adipocyte-derived EVs carry signaling molecules, fatty acid substrates, and metabolic enzyme complexes, inducing mitochondrial metabolic transformation and enhancing the migratory ability of target tumor cells. When obesity is present, the impact of EVs on inter-system metabolic adaptation becomes more significant [160,161]. A ‘candidate mediator’ framing is often more appropriate when discussing exosomal miRNA-driven phenotypes. Reported directional miRNA changes can vary substantially with vesicle source, isolation methods, and quantification platforms; therefore, source tracing and functional perturbation experiments are needed to establish causality [162,163]. In clinical translation, EV-related biomarkers (such as exosomal miRNA and radiotherapy response) can be studied, but their exact association with obesity requires stratification by body composition/metabolic status and validation of tissue source [164]. In summary, any inference about the mechanism of extracellular vesicles (EVs) must adhere to the characterization and reporting standards of MISEV2018 to avoid misinterpreting co-isolation contamination as vesicle-specific function [162]. If EVs are indeed the main promoting factor of obesity-induced cancer, then in experiments between obese and non-obese control models, inhibiting EV release or blocking their uptake should selectively reduce tumor mitochondrial adaptability and invasive behavior, rather than merely reducing inflammation in a general way.

### 4.4. Remodeling of Cell Death Forms: Ferroptosis and Autophagy

Alterations in lipid droplet content and membrane lipid composition associated with fat may increase lipid peroxidation stress, theoretically increasing the possibility of ferroptosis activation [165,166]. However, this does not necessarily mean that tumors are more easily cleared by ferroptosis. One of the factors controlling ferroptosis sensitivity is the PUFA-PL infrastructure activated by ACSL4, which usually leads to a larger pool of oxidizable substrates and higher vulnerability rather than natural resistance [167]. Theorists attribute the resistance of tumors to ferroptosis to two factors: the rapid development of the antioxidant system and the activation of intracellular lipid storage and membrane modification pathways, which can protect the cell membrane from oxidative stress damage [168]. We should study ferroptosis within the “chassis-defense-environmental stress” framework. The impact of ferroptosis on the immune system is not linearly presented as a simple benefit or harm: ferroptosis of tumor cells inhibits the maturation of dendritic cells and antigen cross-presentation, thereby reducing the activity of the adaptive immune system [169]. In the Kras-driven pancreatic tumor model, ferroptosis-related oxidative damage can trigger STING-dependent inflammatory cascades and promote tumorigenesis [170]. If obesity-related lesions simultaneously exhibit high baseline ACSL4 expression, enhanced GPX4/GSH defense capacity, and enhanced STING inflammatory signaling pathways, then inducing ferroptosis alone may be more conducive to enhancing immunosuppression or stimulating inflammatory responses. This model should be evaluated to determine the effectiveness of activating ferroptosis in combination with defense inhibition or normalization of the immune axis. In overweight individuals with metabolic-related fatty liver disease (NAFLD/NASH), the autophagy and lysosomal systems do not function properly, thus the body is unable to clear lipid droplets and damaged mitochondria. This further elevates oxidative stress and inflammation levels, promoting disease progression and potentially creating a microenvironment conducive to cancer development [171]. However, the role of autophagy in tumors varies with the tumor stage. In the early stage of tumors, insufficient autophagy leads to protein aggregation, elevated reactive oxygen species (ROS) levels, and accumulation of damage signals. During the tumor growth stage, some tumors can utilize autophagy to resist hypoxia and nutrient fluctuations, adopting a stress-supportive survival strategy [172]. Autophagy is context-dependent rather than uniformly ‘beneficial’ or ‘harmful’. Mechanistic studies should therefore rely on autophagy-flux measurements (e.g., LC3-II turnover with and without lysosomal inhibitors) together with complementary readouts such as p62/SQSTM1, ULK1–mTOR signaling, and lysosomal acidification and hydrolytic capacity [173]. Autophagy dysfunction has been repeatedly observed in high-fat diet-induced fatty liver models, indicating that autophagy plays a role in the common development of lipid metabolism disorders, inflammation, and fibrosis [174], with fibrosis being a specific entry point in the metabolic–immune–cell death framework. When tumor cells rely on cytoprotective autophagy, pharmacologic or genetic autophagy inhibition can sensitize tumors to therapy in preclinical studies [175,176]. Conversely, when defective autophagy contributes to tissue injury or tumor initiation, further autophagy suppression may worsen damage and inflammation, highlighting the need for context-specific strategies [177].

### 4.5. Life Cycle Scale: Early Developmental Imprinting and Adult Cancer Risk

Obesity, as a systemic exposure, does not confine its carcinogenic effects to metabolic disorders in adulthood but may also shape long-term epigenetic and immune set points through the nutritional-hormonal environment during the developmental period, thereby influencing cancer susceptibility and treatment response windows in adulthood. Relevant reviews have emphasized that fetal/perinatal exposures can leave a sustainable risk “background noise” at the multi-organ level [178]. At the population level, the association between early body fat or growth trajectories and adult tumor risks (such as colorectal cancer) also suggests the need to incorporate the “exposure time window” into risk stratification, rather than relying solely on a single BMI measurement [179].

## 5. Immunooncology and Mechanistic Insights into the “Obesity Paradox”

In the era of immunotherapy, the relationship between obesity and cancer survival rates has undergone a dramatic reversal. A deep understanding of this paradox is crucial for formulating personalized treatment strategies.

### 5.1. The Phenomenology of Paradox

The ‘obesity paradox’ is often operationalized as an association between higher BMI and improved PFS/OS in subsets of patients with advanced cancer. This association is frequently reported in cohorts treated with PD-1/PD-L1 blockade and appears weaker or absent in chemotherapy-only cohorts, although findings vary by tumor type and study design [180,181,182]. However, this is not a universal rule but a contextual effect that depends on cancer type, treatment line, gender, and baseline characteristics. According to a multi-cohort study of metastatic melanoma, obesity is associated with prolonged survival, but the treatment modality and gender stratification show differences [182]. In pooled analyses of ICI-treated cohorts, higher BMI categories have been reported to be independently associated with improved survival after multivariable adjustment; however, this should be interpreted as an observational association rather than a causal effect of BMI per se [181]. The same pattern was also recorded in a multi-center real-world cohort study, but the degree of influence varies depending on the cohort design [183].

A more rigorous explanation is that obesity can reshape the immune metabolism and T-cell exhaustion axis, enhancing PD-1 pathway dependency, thereby increasing the reversibility of PD-1/PD-L1 blockade in specific contexts [30]. The obesity paradox needs to be clarified through precise bias control methods. The biological impact of weight on body composition should not be ignored, as sarcopenic obesity, skeletal muscle mass/density, and visceral fat ratio all independently affect prognosis, and the biological effects of these factors are stronger than BMI. Researchers should use computed tomography (CT) body composition data for stratification or model building as much as possible [184]. Secondly, other factors such as smoking and chronic obstructive pulmonary disease are closely related to weight, intra-body inflammation, tumor mutational burden (TMB), and the efficacy of immune checkpoint inhibitors (ICI), and form easily identifiable epigenetic links in cancer cases such as non-small cell lung cancer (NSCLC) [185]. Thirdly, the pathways of weight loss and cachexia in advanced patients can produce a reverse causal effect, where more severe disease leads to weight loss and poorer treatment outcomes, making the prognosis of the high BMI group appear better [186]. Finally, the combined effects of inflammatory and nutritional markers (C-reactive protein, albumin, neutrophil-to-lymphocyte ratio), treatment frequency, PD-L1 and TMB grouping, drug dosage, and pharmacokinetic exposure (weight and constant dose) will systematically affect the efficacy assessment [187]. To evaluate the statistical basis of this paradox, researchers must construct multivariate models that include these variables and incorporate propensity score weighting, matching, or stratification analysis, which is a necessary prerequisite for determining whether the “paradox” is a statistical illusion (Table 2).

### 5.2. Core Mechanisms: Metabolic Exhaustion vs. Antigenic Exhaustion

To reconcile ‘baseline immunosuppression but greater ICI sensitivity’ in obesity, it is useful to distinguish two modes of T-cell dysfunction. One is metabolic insufficiency/exhaustion driven by nutrient and oxygen stress [188,189,190]; the other is antigen-driven exhaustion with more stable transcriptional and epigenetic programming [191]. The former is more like a “downregulation of function due to metabolic braking” and theoretically has higher plasticity; the latter is more likely to enter the TOX/NR4A-driven exhaustion lineage and form more stable chromatin constraints, thereby limiting the upper limit of reactivation [192,193,194]. Therefore, it is not rigorous to simply equate “PD-1 upregulation” with “irreversible exhaustion” in mechanism descriptions; the key lies in determining whether the driving force behind it is metabolic stress or chronic antigenic stimulation.

Among obesity-associated signals, the leptin–leptin receptor (LepR) axis is a plausible mechanistic link between obesity and PD-1-related T-cell dysfunction. Across multiple tumor models and human datasets, obesity has been associated with PD-1-mediated T-cell dysfunction (partly leptin-driven) yet can coincide with enhanced responses to PD-1/PD-L1 blockade [30]. Clinically, higher BMI has also been linked to better outcomes in immune checkpoint–treated metastatic melanoma cohorts, with substantial heterogeneity across subgroups [182]. The upregulation of PD-1 is not necessarily directly induced by leptin; it may also be indirectly driven by systemic inflammatory factors, tumor antigen load, lipid metabolism disorders, and myeloid cell networks. Therefore, a more prudent statement would be that the leptin axis is more like a background factor that increases the probability of checkpoint dependence rather than a single determining factor. For the view that “PD-1 acts as a metabolic brake” to hold, it must be grounded in an operational discrimination framework. PD-1 engagement can reprogram activated T cells away from efficient glycolysis and amino-acid metabolism and toward a more fatty-acid-oxidation-biased state [195]. Functionally, this can be described as ‘signaling is present and a brake is engaged, but the system is not completely shut down.’ A practical approach is to pair marker panels with functional readouts. Metabolic stress states can present with PD-1 upregulation alongside constrained metabolic flux and mitochondrial stress/remodeling [190,196]. Antigen-driven exhaustion is more closely linked to TOX/NR4A-centered programs and stable epigenetic states; additional markers (e.g., TCF1) should be interpreted together with functional assays and clonal dynamics [197]. These discriminations should be verified in combination with the “pre- and post-treatment change amplitudes” of T-cell receptor (TCR) clonal expansion, mitochondrial mass/membrane potential, oxygen consumption rate/extracellular acidification rate (OCR/ECAR) and cytotoxic molecules (GZMB/PRF1). If obesity-related suppression is more reversible and therefore more ICI-sensitive, this should translate into measurable longitudinal signatures. After treatment, the obese subgroup would be expected to show stronger TCR clonal expansion, recovery of effector programs, and metabolic reactivation (e.g., improved mitochondrial membrane potential and respiratory capacity, and greater glycolytic flexibility). In contrast, if the dominant obstacle comes from the epigenetic fixation of antigenic exhaustion, even if PD-1 is blocked, only limited functional recovery may be achieved and a “recovery ceiling” may be presented. Therefore, reversibility is not an assumed premise but a testable conclusion; it requires the joint support of pre- and post-treatment paired single-cell transcriptome/T-cell receptor sequencing (TCR-seq) and metabolic readouts, and must be validated in different cancer types and different body composition subgroups. Overall, obese tumors may carry a higher baseline metabolic-inflammatory burden and immunosuppressive features. In some contexts, this suppression may be disproportionately mediated through the PD-1 axis, leaving more ‘reversible headroom’ for PD-1/PD-L1 blockade. This framework does not argue that obesity is universally beneficial. Instead, it offers a clinically actionable prediction: when systemic metabolic constraints are alleviated and there is sufficient immune reserve, ICIs may open a ‘reactivation-sensitive window’. Conversely, when reverse causality, catabolic failure, or fixed exhaustion programs predominate, the apparent paradox would be expected to attenuate or reverse. Because the obesity–ICI association is cancer-specific and highly vulnerable to reverse causation and body-composition confounding, Table 3 summarizes representative clinical signals together with the key biases that should be controlled in stratified analyses.

### 5.3. Structured Immunity: Tertiary Lymphoid Structures (TLS) in the Obesity–ICI Context

The traditional view of immunosuppression in obesity holds that it leads to T cell dysfunction [207]. Paradoxically, however, the chronic inflammation caused by obesity can produce chemokines that, under certain conditions, promote the formation of new tertiary lymphoid structures (TLS). TLS are ectopic lymphoid organs located at or within tumor boundaries, containing mature B cell follicles (Germinal Centers) and T cell zones. They act as local “immune factories”, responsible for in situ germinal B cell responses, plasma cell differentiation and antibody production, while also facilitating antigen presentation and clonal expansion of T cells [208,209]. Recent studies on hepatocellular carcinoma (HCC) and renal cell carcinoma have shown that obesity suppresses the systemic immune system, but the lipid-rich environment within the tumor can promote the formation of TLS. Therefore, the obesity paradox may be due to the chronic inflammation associated with obesity and local immune organization, which may increase the probability of TLS occurrence in some tumors and thereby alter the supply of effector cells for immune checkpoint inhibitors (ICI). Importantly, TLS are not obesity-specific and have been described across multiple solid tumors [208,209,210]. Their prevalence and maturity are tumor- and cohort-dependent and should not be generalized as being absent in lean or cachectic patients. In clinical practice, TLS are most reliably assessed by pathology (H&E with immunohistochemistry), while imaging-based inference remains investigational. For example, a multicenter retrospective MRI radiomics model in hepatocellular carcinoma predicted intratumoral TLS status with external validation, supporting feasibility in a tumor-specific setting but requiring prospective validation and standardization before broader clinical deployment [211].

## 6. Clinical Practice of Metabolic Oncology: From Weight-Loss Drugs to Synergistic Immunotherapy

Building on Chapters 2–5, we now move from mechanism to clinical action. With incretin-based weight-loss drugs entering routine care, metabolic intervention becomes a modifiable exposure that may shape cancer risk, progression, and ICI outcomes. A key clinical challenge is that BMI alone rarely indicates whether ICI failure or intolerance is driven predominantly by systemic metabolic/endocrine pressure (the “chassis”), by local exclusionary barriers in the tumor microenvironment (the “execution layer”), or by both. Accordingly, Section 6.1 introduces a chassis–barrier–window workflow to guide biomarker-based stratification and sequencing, and we then map this logic onto therapeutic options—using GLP-1RAs as the reference class for metabolic resetting.

### 6.1. Chassis–Barrier–Window: A Biomarker-Guided Clinical Pathway

In obesity-associated tumors, BMI alone often fails to explain poor tolerance or resistance to immune checkpoint inhibitors (ICIs): whether the dominant driver is sustained systemic metabolic/endocrine stress, local physical and immunological exclusion within the tumor microenvironment (TME), or their combination. Based on the “Systemic Carcinogenic Chassis → TME Executive Barrier” framework, we propose shifting assessment from a static “weight phenotype” to two actionable axes: a quantifiable risk burden and the available actionable room for intervention, thereby translating mechanistic heterogeneity into a practical clinical pathway.

Stratify. Using the dominant driver as a pragmatic handle—while emphasizing a continuum rather than a strict dichotomy—patients can be positioned as Systemic Chassis-Dominant, TME Barrier-Dominant, or Dual-Layer High. Systemically, prioritize proxies for insulin–IGF axis availability (e.g., insulin-driven IGFBP-1/2 suppression patterns and surrogate estimates of free/bioavailable IGF) [13]. Combine these with adipokine balance (e.g., leptin/adiponectin ratio) and systemic inflammatory tone, and interpret them alongside obesity-associated PD-1-dependent immune phenotypes [30]. Within the TME, focus on execution-layer barriers to immune infiltration and therapeutic access: (i) collagen-rich ECM features associated with resistance to PD-1/PD-L1 blockade [105], (ii) lipid-metabolic myeloid programs including TREM2/LPL/CD36 pathways [109], and (iii) NET-associated metastatic programs such as the NET-DNA–CCDC25 axis [119]. In parallel, a longitudinally testable window assessment is formed by integrating checkpoint-dependence clues with exhaustion/suppression states and TLS presence/maturity into traceable decision evidence.

Practical note. Systemic chassis biomarkers are largely blood-measurable today (e.g., leptin/adiponectin ratio, IGF-related proxies, inflammatory markers), whereas many barrier-layer features currently rely on pathology/imaging surrogates and are often trial-enriched (e.g., stromal stiffness/fibrosis, myeloid programs, NET axes). We therefore label biomarkers as clinically deployable versus investigational.

Select. Clinically available levers include GLP-1RAs, metformin, and aromatase inhibitors (context-dependent), while investigational options such as LDHA inhibition or p300/CBP blockade are framed as hypothesis-driven combinations with explicit evidence grading. Following the principle of “address the dominant driver first, then sequence combinations,” patients with systemic chassis dominance and high reversibility are prioritized for metabolic/endocrine resetting (with GLP-1-related drugs as clinical reference levers for tractable exposure modification) [212,213], using systemic inflammation readouts as pharmacodynamic interfaces [214]. For TME barrier dominance, resources are concentrated on execution-layer unlocking: softening/loosening the ECM and incorporating myeloid reprogramming (e.g., anti-TREM2) in combination with ICIs to enhance PD-1/PD-L1 blockade sensitivity [109]. Neuro-immune relief (e.g., β-blockade) should be framed as a timing- and population-dependent exploratory strategy given heterogeneous clinical evidence [215], whereas “metabolic–epigenetic” targets such as LDHA inhibition and p300/CBP blockade are better positioned as hypothesis-driven exploratory combinations with explicit evidence grading [73,74].

Monitor–Iterate (measure–act–remeasure). We emphasize using a consistent marker set throughout the pathway for both selection and pharmacodynamic readback. If systemic indicators improve while stiffness/myeloid/NET modules remain dominant, escalation should proceed sequentially—Barrier Removal → Effector Introduction (ICI) → Scar/Residual-Risk Management—rather than parallel stacking, forming a reproducible “Measure–Act–Remeasure” loop.

### 6.2. GLP-1 Receptor Agonists: From Metabolic Regulation to Immune Remodeling

As highlighted in the stratification framework, addressing the systemic chassis is the first priority for metabolically driven tumors. While metformin remains a foundational metabolic therapy, GLP-1 receptor agonists represent a stronger lever for weight-loss-linked immune remodeling. With incretin-based therapies entering routine care, evidence up to 2025 (including RCT meta-analyses and some real-world studies) does not suggest a clear carcinogenic risk signal for GLP-1RAs. Early rodent concerns (medullary thyroid carcinoma and pancreatic cancer) were not supported in pooled human RCT analyses, but longer follow-up is still needed because cancer events are rare and latency can be long [212,213]. Real-world cohort data suggest that GLP-1RA use is associated with lower incidence of endometrial, ovarian, and meningioma cancers, while a borderline signal has been reported for kidney cancer. These observational findings imply cancer-type heterogeneity and warrant attention to residual confounding and indication bias [214].

Phase III DREAMS-1 and DREAMS-2 trials place the GCGR/GLP-1R dual agonist mazdutide in a combined ‘glycemic control + weight loss’ evidence framework. DREAMS-1 (placebo-controlled) reported significant HbA1c reduction and dose-dependent weight loss at 24 weeks, whereas DREAMS-2 (active comparator) reported superiority over dulaglutide in both glycemic control and body-weight reduction [215,216]. It should be emphasized that these endpoints do not address tumor occurrence or immune treatment outcomes; thus, their significance for “carcinogenic risk/anti-tumor immunity” should be regarded as indirect evidence. In principle, deeper correction of adiposity burden and reduction in inflammatory baseline may lower the pro-tumor niche and the immune-toxicity “background noise,” but longer follow-up and tumor-cohort validation are still needed [217,218].

Mechanistically, the systemic anti-inflammatory effect of GLP-1RAs (e.g., decreased hsCRP) may constitute a translatable interface for shifting the threshold of anti-tumor immunity [217]. At the cellular level, one hypothesis is partial reversal of iNKT dysfunction: invariant natural killer T (iNKT) cells often decrease or become functionally exhausted in overweight individuals, and GLP-1RA treatment may alter their distribution and cytokine output, thereby improving immune surveillance [219,220]. Additionally, GLP-1RAs have been reported to inhibit the NLRP3 inflammasome in macrophages and reduce IL-1β production, weakening a pro-inflammatory fuel supply for the tumor milieu [221]. Based on this metabolism–immunity coupling logic, clinical trials are evaluating GLP-1RA as an adjuvant strategy for ICI to enhance responses to PD-1 antibodies and reduce irAE risk in both obese and non-obese patients. However, at this stage, it is more appropriate to position GLP-1RA as a candidate for “next-generation metabolic concomitant therapy,” rather than equating it with an intervention that has already demonstrated anti-tumor outcomes [218].

### 6.3. New Strategies Targeting the Microenvironment

While metabolic drugs address systemic drivers, resistance often persists because local physical and immune barriers remain. In our ‘TME Executive Barrier’ model, we group candidate combinations into three actionable axes [222,223]. First, stiffness-targeting approaches (e.g., inhibiting LOX-mediated collagen cross-linking or integrin/adhesion signaling) may soften tumors, improve immune-cell trafficking, and enhance responses to PD-1 blockade in preclinical models [105,224,225]. Second, beta-blockade aims to dampen sympathetic-driven immunosuppression and metastasis; early clinical and preclinical data are promising but heterogeneous, and a recent meta-analysis suggests that benefit may depend on patient selection and timing [226,227,228]. Third, given the persistence of post-weight-loss transcriptional/epigenetic ‘scars’, epigenetic interventions have been proposed as a longer-term strategy, but immune effects and off-target toxicities require careful evaluation [91]. Overall, a sequential logic—‘barrier removal → effector introduction → memory/residual-risk management’—may be preferable to stacking all interventions in parallel.

## 7. Conclusions and Outlook: Towards a New Era of “Metabolic-Immunotumorology”

The research on the relationship between obesity and cancer is at a critical juncture of paradigm shift. As reviewed in this article, we no longer merely view obesity as a “caloric reservoir” of energy excess, but redefine it as a systemic carcinogenic state that reshapes the neuro-immune network, alters the physical microenvironment, and disrupts inter-organ communication. The development of obesity-related tumor treatment can be summarized into three main areas. 1. The impact of obesity-induced fibrotic extracellular matrix. Reducing mechanical memory and residual risk. Therefore, risk management should not only focus on restoring normal weight but also be based on the research of mechanical-targeted drugs to eliminate the epigenetic scars remaining at the physical level. 2. Reversible myeloid module remodeling. The main focus should be on the metabolic myeloid module based on TREM2+/LAM-like macrophages, utilizing their coupled functions in lipid response and T cell rejection as the primary target and companion diagnostic marker for combined immune checkpoint inhibitors (ICIs). 3. Precise metabolic-epigenetic targeting. Obesity-induced lactic acid stress leads to histone lactylation, thereby altering the fate of T cells and causing them to express more PD-1. It is suggested that future research should adopt spatial causal experiments and metabolic tracing techniques to turn this logical reasoning chain into a clinically verified causal relationship. Future treatments should involve interactions between systems. By incorporating metabolic interventions, including GLP-1 receptor agonists, into the framework of immune remodeling and organizing combined treatments based on the logic of gradually eliminating obstacles, delivering effector drugs, and finally managing residual risks, the relationship between obesity and tumors will be transformed into a true multi-level treatment approach.

## Figures and Tables

**Table 1:** Systemic driver factors and quantifiable biomarkers.

Driver Module	Molecular Marker(s)	Direction of Change in Obesity	Key Oncogenic Output	Clinical Leverage Point	Representative Evidence
Pro-growth axis	Circulating bioavailable/free IGF-1	↑ (often via insulin-suppressed IGFBP-1/2; total IGF-1 may be variable)	PI3K–AKT–mTOR activation and mitogenic signaling	Metformin; insulin sensitization	[64,65,66,67]
Adipokine axis	Leptin/Adiponectin ratio	Markedly imbalanced (leptin ↑, adiponectin ↓)	PD-1-linked exhaustion-like state (metabolic/pseudo-exhaustion phenotype) and checkpoint dependence in obesity contexts	GLP-1R/GCGR co-agonists (weight-loss metabolic reset)	[30,68,69,70,71]
Steroid microenvironment	Aromatase (CYP19A1)	↑ local expression/activity (especially in adipose stromal compartments post-menopause)	Local estrogen “bath” fueling hormone-dependent tumor growth	Aromatase inhibitors (AIs); anti-inflammatory intervention	[40,41,72]
Metabolic epigenetics	H3K18la (histone lactylation)	↑ accumulation (lactate-coupled)	Transcriptional program stabilization/”locking” (e.g., immune evasion/drug resistance–permissive states)	Exploratory (preclinical): lactate/LDH-axis modulation and p300/CBP inhibition; not clinically approved for this indication	[73,74,75,76]

**Table 2:** Bias map for “obesity paradox” signals in ICI cohorts and analytic remedies.

Bias/Confounder Type	Mechanism & Impact on the “Obesity Paradox”	Analytic Remedies & Mitigation Strategies
Reverse Causation (Cachexia/Sarcopenia)	Severe or rapidly progressing disease causes pre-treatment weight loss. These patients have lower BMI and poorer OS, making the high-BMI group artificially appear to have a survival advantage.	Stratify by CT-based body composition (e.g., sarcopenia). Adjust for cachexia biomarkers (e.g., albumin, CRP, NLR). Exclude early deaths (e.g., within 3–6 months) in landmark analyses.
Collider/Selection Bias	Conditioning the cohort on “having advanced cancer” or “receiving ICI” can induce spurious associations between two independent risk factors (e.g., obesity and smoking) that both influence selection into the cohort.	Construct Directed Acyclic Graphs (DAGs) to identify colliders. Perform sensitivity analyses across different selected populations. Avoid adjusting for variables that are consequences of both obesity and tumor progression.
Immortal Time Bias (Common in real-world data)	Defining “obesity” or “treatment exposure” based on post-baseline weight changes or cumulative doses guarantees the patient survived long enough to be classified, artificially inflating survival in that group.	Ensure strict time-zero alignment (e.g., BMI strictly at ICI initiation). Use time-dependent covariate modeling. Conduct landmark analyses.
Confounding by Smoking/COPD	Smokers often have lower BMI but higher Tumor Mutational Burden (TMB), which drives better ICI responses in cancers like NSCLC, creating a complex confounding triangle.	Stratify models by smoking status and pack-years. Adjust directly for TMB or PD-L1 status where available. Propensity score (PS) matching or weighting.
Dosing/Pharmacokinetic (PK) Artifacts	Flat (fixed) vs. weight-based dosing strategies may result in different pharmacokinetic exposures or clearance rates in obese vs. lean patients.	Adjust for dosing strategy and relative dose intensity. Incorporate PK/pharmacodynamic exposure variables into multivariable models.

**Table 3:** Representative ICI cohort signals and key bias considerations (qualitative summary).

Cancer Type	Obesity Definition (BMI)	Reported ICI Outcome Signal (ORR/PFS/OS)	Key Mechanistic Interpretation (Hypothesis-Level)	Potential Bias/Confounding to Flag	Evidence (Representative)
Non-small cell lung cancer (NSCLC)	≥30 kg/m^2^	Multiple cohorts/trial-level analyses report improved survival metrics in higher BMI groups treated with PD-(L)1 blockade	Checkpoint dependence may be enriched in specific immune states; apparent benefit can reflect reversibility of metabolic braking in a subset	Smoking history; performance status; reverse causation (pre-treatment weight loss/cachexia); body composition (sarcopenia/sarcopenic obesity)	[181,198,199]
Melanoma	≥30 kg/m^2^	Several cohorts/meta-analyses report an “obesity paradox” signal (often sex- and inflammation-dependent)	Leptin/LepR-linked nutrient-excess signaling may increase PD-1-associated inhibitory tone while preserving reactivation potential in some contexts	Sarcopenia/sarcopenic obesity; sex effects; systemic inflammation as a modifier	[182,200,201,202]
Renal cell carcinoma (RCC)	≥30 kg/m^2^ (some use ≥ 25)	Higher BMI frequently associates with better outcomes in ICI-treated cohorts/meta-analyses; heterogeneity across regimens and risk strata	Tumor immune contexture matters; TLS can stratify responsiveness to PD-1 blockade (not obesity-specific, but relevant to interpretation)	Reverse causation (cachexia); IMDC risk; comorbidities; body composition metrics	[203,204,205,206]

## Data Availability

No new data were created or analyzed in this study.
